# Novel pentafluorosulfanyl-containing triclocarban analogs selectively kill Gram-positive bacteria

**DOI:** 10.1128/spectrum.00071-24

**Published:** 2024-05-03

**Authors:** Ali Pormohammad, Melika Moradi, Josefien W. Hommes, Eugènia Pujol, Lieve Naesens, Santiago Vázquez, Bas G. J. Surewaard, Mohammad Zarei, Manuel Vazquez-Carrera, Raymond J. Turner

**Affiliations:** 1 Department of Biological Sciences, Faculty of Science, University of Calgary, Calgary, Alberta, Canada; 2 MHCombiotic Inc., Calgary, Alberta, Canada; 3 Department of Microbiology, Faculty of Medicine, Ahvaz Jundishapur University of Medical Science, Ahvaz, Iran; 4 Calvin, Phoebe and Joan Snyder Institute for Chronic Diseases, Cumming School of Medicine, University of Calgary, Calgary, Alberta, Canada; 5 Laboratori de Química Farmacèutica (Unitat Associada al CSIC), Facultat de Farmàcia i Ciències de l’Alimentació, Universitat de Barcelona, Barcelona, Spain; 6 Institute of Biomedicine of the University of Barcelona (IBUB), University of Barcelona, Barcelona, Spain; 7 Rega Institute for Medical Research, KU Leuven, Leuven, Belgium; 8 Renal Division, Brigham & Women’s Hospital, Harvard Medical School, Boston, Massachusetts, USA; 9 John B. Little Center for Radiation Sciences, Harvard T. H. Chan School of Public Health, Boston, Massachusetts, USA; 10 Pharmacology Unit, Faculty of Pharmacy and Food Sciences, University of Barcelona, Barcelona, Spain; 11 Pediatric Research Institute-Hospital Sant Joan de Déu, Esplugues de Llobregat, Barcelona, Spain; 12 Spanish Biomedical Research Center in Diabetes and Associated Metabolic Diseases (CIBERDEM)-Instituto de Salud Carlos III, Madrid, Spain; Duke University, Durham, North Carolina, USA

**Keywords:** novel antimicrobials, Gram-positives, *Staphylococcus aureus*, *MRSA*, pentafluorosulfanyl, diarylurea scaffold, antibiofilm, antibacterial

## Abstract

Novel antimicrobial agents are needed to combat antimicrobial resistance. This study tested novel pentafluorosulfanyl-containing triclocarban analogs for their potential antibacterial efficacy. Standard procedures were used to produce pentafluorosulfanyl-containing triclocarban analogs. Twenty new compounds were tested against seven Gram-positive and Gram-negative indicator strains as well as 10 clinical isolates for their antibacterial and antibiofilm activity. Mechanistic investigations focused on damage to cell membrane, oxidizing reduced thiols, iron-sulfur clusters, and oxidative stress to explain the compounds’ activity. Safety profiles were assessed using cytotoxicity experiments in eukaryotic cell lines. Following screening, selected components had significantly better antibacterial and antibiofilm activity against Gram-positive bacteria in lower concentrations in comparison to ciprofloxacin and gentamycin. For instance, one compound had a minimum inhibitory concentration of <0.0003 mM, but ciprofloxacin had 0.08 mM. Mechanistic studies show that these novel compounds do not affect reduced thiol content, iron-sulfur clusters, or hydrogen peroxide pathways. Their impact comes from Gram-positive bacterial cell membrane damage. Tests on cell culture toxicity and host component safety showed promise. Novel diarylurea compounds show promise as Gram-positive antimicrobials. These compounds offer prospects for study and optimization.

**IMPORTANCE:**

The rise of antibiotic resistance among bacterial pathogens poses a significant threat to global health, underscoring the urgent need for novel antimicrobial agents. This study presents research on a promising class of novel compounds with potent antibacterial properties against Gram-positive bacteria, notably *Staphylococcus aureus* and *MRSA*. What sets these novel analogs apart is their superior efficacy at substantially lower concentrations compared with commonly used antibiotics like ciprofloxacin and gentamycin. Importantly, these compounds act by disrupting the bacterial cell membrane, offering a unique mechanism that could potentially circumvent existing resistance mechanisms. Preliminary safety assessments also highlight their potential for therapeutic use. This study not only opens new avenues for combating antibiotic-resistant infections but also underscores the importance of innovative chemical approaches in addressing the global antimicrobial resistance crisis.

## INTRODUCTION

Bacterial resistance to antimicrobials has become a pressing concern in healthcare, demanding the development of effective formulations targeting multi-resistant pathogens (1). To address this challenge, researchers have turned to drug repurposing, a strategy that explores novel uses for authorized or experimental pharmaceuticals beyond their initial applications, offering advantages such as reduced costs and shorter development timelines (2). In this context, the exploration of multitarget molecules has emerged as a promising approach to counteract pathogens through diverse mechanisms. Diarylureas, such as regorafenib, sorafenib, linifanib, ripretinib, and tivozanib, have long been recognized as anticancer agents (3). One intriguing avenue involves the repurposing of diarylureas with anticancer properties for novel indications, such as antimicrobial, anti-inflammatory, and antiviral applications (4).

Trifluoromethyl groups, serving as bio-isosteric replacements for chlorine atoms, have found widespread use in medicinal chemistry. Hence, it is unsurprising that specific N,N′-diarylureas that comprise a trifluoromethyl moiety have demonstrated encouraging antibacterial properties (5). An example is cloflucarban (TFC, 3-trifluoromethyl-4,4′-dichlorocarbanilide), a trifluoromethyl-substituted diarylurea that shares a similar spectrum of activity and pharmacokinetic profile with triclocarban (TCC). In recent times, a number of diarylureas analogs of TCC have been identified as having antibacterial and antifungal properties. The presence of pentafluorosulfanyl and trifluoromethyl coumarin groups characterizes these analogs. Given the risks associated with TCC, the need for alternative antimicrobial agents has become crucial (3).

In recent years, a novel bio-isosteric trifluoromethyl unit, the pentafluorosulfanyl group (SF_5_), has emerged in medicinal chemistry, finding applications in agriculture and material chemistry (6–8). Regarded as a “super-trifluoromethyl group,” the SF_5_-group possesses several advantageous properties over its isostere trifluoromethyl group, the compound exhibits a tetragonal bipyramidal morphology and possesses a greater electronegativity value of 3.65, in comparison to trifluoromethyl’s value of 3.36. Additionally, it displays higher lipophilicity and notable steric volume, which is a bit smaller compared with *tert*-butyl but more than trifluoromethyl. The compound’s hydrolytic and chemical stability has also been approved (9). These unique characteristics have led to a significant rise in the utilization of SF_5_ in medicinal chemistry over the past decade, making it an exceptionally appealing substitute for medicinal applications. SF_5_-containing building blocks have garnered attention among medicinal chemists owing to their stated ability to decelerate metabolic rates and their eco-friendliness vis-à-vis the lack of chlorine atoms, despite their elevated cost relative to analogous CF_3_ compounds. Building upon the growing utilization of SF_5_ in medicinal chemistry and its favorable environmental profile, the objective of this study was to incorporate this innovative group onto the N,N′-diarylurea scaffold to explore novel antimicrobial agents (10).

This present study aims to explore the synthesis and antimicrobial and antibiofilm activity of novel diphenylurea agents, particularly inspired by TCC but bearing different aryl moieties. Mechanistic studies, such as cell membrane or hydrogen peroxide assays, are conducted to elucidate the compounds’ modes of action. Cytotoxicity assays using cell lines will ensure their safety as a unique class of antimicrobials.

## RESULT AND DISCUSSION

### Chemistry

**Fig 1:**
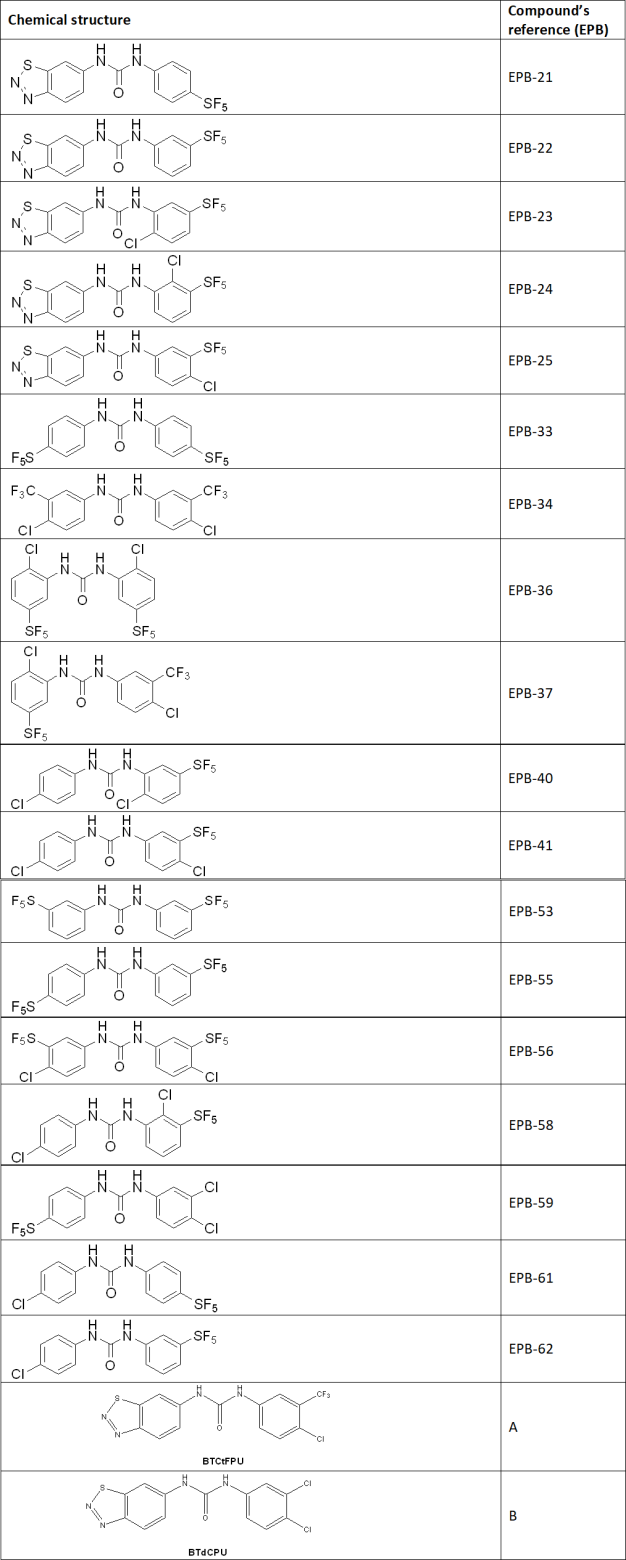
Compounds EPB-21, EPB-22, EPB-23, EPB-24, EPB-25, and EPB-36 (11) and compounds EPB-33, PB-34, EPB-37, EPB-40, EPB-41, EPB-55, EPB-56, EPB-58, EPB-59, EPB-61, EPB-62 (5, 11), and EPB-53 have been published in our previous works (5, 12).

The 18 *N,N’*-diarylureas evaluated in this work were synthesized following a simple and straightforward procedure consisting of the coupling of phenyl isocyanates with the corresponding anilines, as previously reported by some of us (5, 11, 12) (Fig. 1). In turn, the intermediate phenyl isocyanates were either commercially available or synthesized *in situ* from the reaction of the precursor anilines with triphosgene. The analytical data of the compounds fully agreed with the data previously reported (5, 11, 12).

### Antibacterial activity

The N,N’-diarylurea chemotype has been extensively studied in various published works, particularly regarding its anticancer and antischistosomal activities (11, 13, 14). Consistent with earlier findings, TCC and cloflucarban demonstrated antibacterial action against Gram-positive bacteria; however, no activity against Gram-negative pathogens was found (15). Similarly, no antibacterial activity was demonstrated by the novel analogs produced in this work against the Gram-negative pathogens *Escherichia coli* and *Pseudomonas aeruginosa*. However, the *Staphylococcus* genera included in this work (*S. aureus*, *Staphylococcus epidermidis*, and an methicillin-resistant *S. aureus* (*MRSA)* clinical isolate) were the only ones in which TCC and cloflucarban showed activity against Gram-positive bacteria. It is important to note that the majority of the newly designed pentafluorosulfanyl derivatives demonstrated a broader spectrum of antimicrobial activity than TCC and cloflucarban (5). It is noteworthy that our compounds are new and that, except from our investigations, no publication has been written explicitly discussing these special chemicals. We explored the antibacterial activity of compounds against several bacterial pathogens, obtaining measurements of minimum inhibitory concentration (MIC) and minimum bactericidal concentration (MBC) (Table 1). The only exception was the effect of compound **59** on *Proteus mirabilis* with MIC and MBC of 0.002 mM. However, regarding the main outcome measure of this study, three compounds, **59**, **61**, and **62**, were effective against *S. aureus*, *S. epidermidis*, and *MRSA* spp. Exact dosages for each compound and bacteria are illustrated in Table 1. Compound **59** showed the best efficacy against *S. aureus*, *MRSA*, and *S. epidermidis*. Compound **61** also was effective against these bacteria with relatively higher doses. Compound **62** was also effective against these Gram-positive bacteria; however, it had the highest doses comparatively (Fig. S1), given the efficacy against the reference strain of *MSRA* we tested. Encouragingly, further testing against 10 clinical isolates of *MRSA* confirmed similarly effective doses of MIC and MBC for compounds **59**, **61**, and **62**, underscoring their potential clinical relevance (Table 2). Notable similarities were found between the results of our earlier investigation and the analysis of clinical isolates (5). Previous investigation discovered that the clinical isolate, *MRSA,* showed almost comparable antimicrobial sensitivity with the laboratory strain, despite clinical isolates being more resistant to antibiotics (5). Additionally, Yunfeng Xie et al. looked into the antibacterial properties of 12 diphenylurea compounds. A number of these substances exhibited a specific antimicrobial activity, mostly targeted toward Gram-positive bacteria. Compounds ZJ-2, ZJ-3, and ZJ-8 stood out in particular because they continuously demonstrated excellent antibacterial effectiveness against 36 drug-resistant strains of Gram-positive bacteria, including *MRSA* and *S. aureus*. These strains had demonstrated resistance to high-gentamicin as well as three or more other antimicrobial drugs. However, it is important to note that these compounds did not exhibit any antibacterial activity against 14 Gram-negative bacterial strains or seven *Candida* strains (16).

**TABLE 1:** MIC, MBC, and minimum biofilm inhibitory concentration (MBIC) of diphenyl-urea antibiotics for *P. aeruginosa*, *S. aureus,* and *E. coli*^*a*^^,^ ^*b*^

		Agents	*E. coli*	*P. aeruginosa*	*K. pneumonia*	*P. mirabilis*	*S. aureus*	MRSA	*S. epidermidis*
MIC (mM)	diphenyl-urea	**EPB-21**	160	310	>5	>5	1,250	2,500	U/A
		**EPB-22**	310	310	>5	>5	0.61	0.61	U/A
		**EPB-23**	310	310	>5	>5	2.4	4.9	U/A
		**EBP-24**	310	160	>5	>5	**0.076**	**0.15**	U/A
		**EBP-25**	310	U/A	>5	>5	9.8	U/A	U/A
		**EBP-33**	310	620	>5	>5	**0.019**	**0.0048**	U/A
		**EBP-34**	310	620	>5	0.625	**0.0038**	**0.0019**	**0.0002**
		**EBP-36**	310	310	>5	>5	**0.076**	**0.61**	U/A
		**EBP-37**	310	310	>5	0.625	**0.0012**	**0.008**	**0.008**
		**EBP-40**	310	310	>5	0.625	0.48	**0.15**	1.2
		**EBP-41**	310	310	>5	>5	**0.038**	**0.0095**	U/A
		**EBP-53**	310	310	>5	>5	1.2	**1.2**	U/A
		**EBP-55**	310	310	>5	>5	**0.019**	**0.0048**	U/A
		**EBP-56**	310	310	>5	>5	4.9	0.30	U/A
		**EBP-58**	310	310	>5	>5	620	20	U/A
		**EBP-59**	310	310	>5	0.002	**<0.0003**	**0.0024**	**0.004**
		**EPB-61**	310	310	>5	>5	**0.0095**	**0.16**	**0.001**
		**EBP-62**	310	310	>5	>5	**0.0003**	**0.038**	**0.02**
		**A**	310	310	>5	>5	2,500	310	U/A
		**B**	310	310	>5	>5	9.8	4.9	U/A
	Antibiotics	**Cip**	0.002	0.061	0.01	0.02	0.08	0.160	1.2
		**Gen**	0.01	0.012	0.001	0.001	0.04	0.98–0.39	0.3
**MBC** (mM)	diphenyl-urea	**EPB-21**	>2,500	1,250	>5	>5	> 2,500	>2,500	U/A
		**EPB-22**	>2,500	2,500	>5	>5	20	4.9	U/A
		**EPB-23**	>2,500	>2,500	>5	>5	20	39	U/A
		**EBP-24**	>2,500	1,250	>5	>5	78	2,500	U/A
		**EBP-25**	>2,500	U/A	>5	>5	78	U/A	U/A
		**EBP-33**	>2,500	1,250	>5	>5	20	2.4	U/A
		**EBP-34**	>2,500	1,250	>5	0.625	**0.01**	**0.061**	**0.01**
		**EBP-36**	2,500	310	>5	>5	78	9.8	U/A
		**EBP-37**	2,500	310	>5	0.625	4.9	**0.04**	**0.04**
		**EBP-40**	2,500	310	>5	0.625	**1.2**	0.61	**0.9**
		**EBP-41**	2,500	310	>5	>5	**1.2**	1.2	U/A
		**EBP-53**	2,500	310	>5	>5	1,250	2,500	U/A
		**EBP-55**	2,500	310	>5	>5	78	**0.15**	U/A
		**EBP-56**	2,500	310	>5	>5	78	**0.61**	U/A
		**EBP-58**	310	>2,500	>5	>5	2,500	620	U/A
		**EBP-59**	310	1,250	>5	0.002	**0.0061**	**0.02**	**0.01**
		**EPB-61**	310	>2,500	>5	>5	**0.095**	**0.30**	**0.04**
		**EBP-62**	310	>2,500	>5	>5	0.30	**0.076**	**0.1**
		**A**	310	1,250	>5	>5	>2,500	620	U/A
		**B**	310	>2,500	>5	>5	620	160	U/A
	Antibiotics	**Cip**	0.002	2.4	0.5	0.8	0.08	0.160	1.2
		**Gen**	0.01	1.2	0.05	0.05	0.04	0.98–0.39	0.3
**MBIC** (mM)	diphenyl-urea	**EPB-21**	310	1,250	>5	>5	620	>2,500	U/A
		**EPB-22**	310	1,250	>5	>5	4.9	20	U/A
		**EPB-23**	310	1,250	>5	>5	4.9	39	U/A
		**EBP-24**	310	1,250	>5	>5	4.9	2,500	U/A
		**EBP-25**	310	U/A	>5	>5	160	u/A	U/A
		**EBP-33**	310	1,250	>5	>5	0.15	**0.15**	U/A
		**EBP-34**	310	1,250	>5	0.625	0.30	**0.076**	**0.001**
		**EBP-36**	**160**	1,250	>5	<5	78	1.2	U/A
		**EBP-37**	>2,500	1,250	>5	0.625	**0.0095**	**<0.0003**	**0.001**
		**EBP-40**	>2,500	1,250	>5	0.625	0.15	0.61	**0.9**
		**EBP-41**	>2,500	1,250	>5	>5	0.30	0.30	U/A
		**EBP-53**	>2,500	1,250	>5	>5	310	1,250	U/A
		**EBP-55**	>2,500	1,250	>5	>5	0.30	**0.15**	U/A
		**EBP-56**	>2,500	1,250	>5	>5	9.8	1.2	U/A
		**EBP-58**	310	1,250	>5	>5	2,500	39	U/A
		**EBP-59**	310	1,250	>5	0.002	**0.019**	**0.30**	**0.002**
		**EPB-61**	310	1,250	>5	>5	**0.0095**	**0.30**	**0.001**
		**EBP-62**	310	1,250	>5	>5	0.38	**0.038**	**0.01**
		**A**	310	1,250	>5	>5	>2,500	620	U/A
		**B**	310	1,250	>5	>5	20	9.8	U/A
	Antibiotics	**Cip**	0.002	2.4	0.01	0.02	0.08	0.160	1.2
		**Gen**	0.01	1.2	0.001	0.001	0.04	0.98–0.39	0.3

^*a*^ 3-(1,2,3-benzothiadiazol-6-yl)-1-(4-pentafluoro-λ^6^-sulfanyl)urea = EPB-21, 3-(1,2,3-benzothiadiazol-6-yl)-1-(3-pentafluoro-λ^6^-sulfanyl)urea = EPB-22, 3-(1,2,3-benzothiadiazol-6-yl)-1-(2-chloro-5-pentafluoro-λ^6^-sulfanyl)urea = EPB-23, 3-(1,2,3-benzothiadiazol-6-yl)-1-(2-chloro-3-pentafluoro-λ^6^-sulfanyl)urea = EPB-24, 3-(1,2,3-benzothiadiazol-6-yl)-1-(4-chloro-3-pentafluoro-λ^6^-sulfanyl)urea = EBP-25, 1,3-bis(4-pentafluoro-λ^6^-sulfanyl)urea = EBP-33, 1,3-di(4-chloro-3-(trifluoromethyl)phenyl)urea = EBP-34, 1,3-bis(2-chloro-5-pentafluoro-λ^6^-sulfanyl)urea = EBP-36, 3-(4-chloro-3-(trifluoromethyl)phenyl)-1-(2-chloro-5-pentafluoro-λ^6^-sulfanyl)urea = EPB-37, 1-(2-Chloro-5-(pentafluoro-λ^6^-sulfanyl)phenyl)-3-(4-chlorophenyl)urea = EPB-40, 1-(4-chloro-3-(pentafluoro-λ^6^-sulfanyl)phenyl)-3-(4-chlorophenyl)urea = EPB-41, 1,3-bis(3-(Pentafluoro-λ^6^-sulfanyl)phenyl) urea= EPB-53, 1-(3-(pentafluoro-λ^6^-sulfanyl)phenyl)-3-(4-(pentafluoro-λ^6^-sulfanyl)phenyl)urea = EPB-55, 1,3-bis(4-Chloro-3-(pentafluoro-λ^6^-sulfanyl)phenyl) urea = EPB-56, 1-(2-Chloro-3-(pentafluoro-λ^6^-sulfanyl)phenyl)-3-(4-chlorophenyl)urea = EPB-58, 1-(3,4-dichlorophenyl)-3-(4-pentafluoro-λ^6^-sulfanyl)phenyl) urea= EPB-59, 1-(4-Chlorophenyl)-3-(4-(pentafluoro-λ^6^-sulfanyl)phenyl)urea = EPB-61, 1-(4-chlorophenyl)-3-(3-(pentafluoro-λ^6^-sulfanyl)phenyl)urea = EPB-62, Ciprofloxacin = Cip, Gentamicin = Gen, Ampicillin = Amp MIC = minimum inhibitory concentration, MBC = Minimum bactericidal concentration, MHB = Mueller Hinton Broth, and MBIC = Minimum biofilm inhibitory concentration, U/A = Unavailable

^*b*^ The **bold values** show that the components are antibacterial/anti-biofilm in lower concentration in comparison to antibiotics.

**TABLE 2:** MIC, MBC, and MBIC of diphenyl-urea antibiotics against *MRSA* clinical isolates^*a*^

MRSA clinical isolates			*MRSA* #1	*MRSA* #2	*MRSA* #3	*MRSA* #4	*MRSA* #5	*MRSA* #6	*MRSA* #7	*MRSA* #8	*MRSA* #9	*MRSA* #10
**MIC** (mM)	diphenyl-urea	**EBP-34**	0.6	<0.002	0.08	<0.002	0.01	0.01	<0.002	0.005	<0.002	<0.002
		**EBP-37**	0.01	<0.002	0.08	<0.002	0.01	<0.002	0.01	<0.002	0.005	<0.002
		**EBP-40**	0.02	0.02	0.08	0.04	0.02	0.04	0.04	0.04	0.02	0.04
		**EBP-59**	<0.002	<0.002	<0.002	<0.002	<0.002	<0.002	<0.002	<0.002	<0.002	<0.002
		**EPB-61**	<0.002	<0.002	<0.002	<0.002	<0.002	<0.002	<0.002	<0.002	<0.002	<0.002
		**EBP-62**	<0.002	<0.002	<0.002	<0.002	<0.002	<0.002	0.005	0.005	<0.002	0.005
	Antibiotics	**Cip**	0.4	0.05	0.4	0.05	0.2	0.2	0.2	1.6	0.4	0.05
		**Gen**	0.1	0.05	0.05	0.05	0.8	3	0.05	0.05	0.05	0.05
**MBC** (mM)	diphenyl-urea	**EBP-34**	<0.002	<0.002	0.08	<0.002	0.01	0.01	0.01	0.005	<0.002	0.01
		**EBP-37**	1.2	0.01	0.6	0.01	0.01	<0.002	0.04	0.01	0.01	0.01
		**EBP-40**	1.2	0.04	0.16	0.16	0.04	0.16	0.08	0.08	0.08	0.01
		**EBP-59**	0.005	<0.002	<0.002	0.005	0.01	<0.002	<0.002	0.005	0.02	0.04
		**EPB-61**	0.04	0.01	0.01	0.02	0.04	0.02	0.02	0.04	0.04	0.02
		**EBP-62**	0.08	0.005	<0.002	0.02	0.02	0.005	0.01	0.08	0.04	0.08
	Antibiotics	**Cip**	1.6	0.4	0.05	0.4	0.4	0.4	0.05	12.5	1.6	0.8
		**Gen**	1.6	0.2	0.05	0.05	6.25	25	1.6	0.05	0.05	0.05
**MBIC** (mM)	diphenyl-urea	**EBP-34**	1.2	<0.002	0.08	<0.002	0.01	0.01	0.02	0.005	<0.002	<0.002
		**EBP-37**	0.02	<0.002	0.3	0.01	0.01	<0.002	0.01	<0.002	<0.002	<0.002
		**EBP-40**	<0.002	<0.002	0.08	<0.002	0.02	0.04	<0.002	<0.002	<0.002	<0.002
		**EBP-59**	<0.002	<0.002	<0.002	<0.002	<0.002	<0.002	<0.002	<0.002	<0.002	<0.002
		**EPB-61**	<0.002	<0.002	<0.002	<0.002	<0.002	<0.002	<0.002	<0.002	<0.002	<0.002
		**EBP-62**	<0.002	<0.002	<0.002	<0.002	<0.002	<0.002	<0.002	<0.002	<0.002	<0.002
	Antibiotics	**Cip**	0.05	0.05	0.2	0.05	0.05	0.2	0.05	0.05	0.05	0.05
		**Gen**	0.05	0.05	0.05	0.05	0.1	12.5	0.05	0.05	0.05	0.05

^*a*^ Bold values are used to highlight the names of the compounds as they are numbers, compared to the antimicrobial number.

The antibiofilm activity of antimicrobials can vary significantly compared with their effectiveness against planktonic forms of bacteria due to differences in physiology and structure of the biofilm (17). In fact, certain reports have demonstrated that biofilms require concentrations of antibiotics up to 100 times higher than those needed to eliminate planktonic bacteria (18–20). To assess the antibiofilm potential of the novel compounds, their MBIC values were determined (Table 1). Similar to planktonic antibacterial activity, compounds **59**, **61**, and **62** were effective against biofilm form of Gram-positive bacteria. Compounds **59** and **61** had the best antibiofilm activity in comparison to other compounds against *S. epidermidis*, *MRSA*, and *S. aureus*. Compound **62** also showed relatively better results against these bacteria. When evaluating these compounds against 10 clinical isolates of *MRSA*, all of these isolates had their biofilms inhibited by these compounds (Table 2). It is highlighted in Dr. Manuel Vazquez-Carrera’s earlier work how important it is to generate innovative approaches for managing pre-existing biofilms on medical equipment, especially catheters. The effectiveness of several chemicals in eliminating biofilms generated in catheters is being evaluated, contrasting their results with those of conventional therapies such as TCC and the antibiotic ciprofloxacin. Compounds 10 and 12 (#**33** and **34** in this study) showed encouraging potential in biofilm management, with similar biofilm clearance rates as TCC within catheters. Moreover, they were almost as effective as ciprofloxacin, suggesting an important development in the fight against biofilm-associated infections in healthcare settings (5).

### Antimicrobial mechanism of action

A wide variety of mechanisms of antimicrobial action are reported for antimicrobial agents. Here, in this study, the most common mechanisms, such as oxidation of the redox buffer reflected in reduced thiol content (18), related oxidative stress as seen by an increase in reactive oxygen species (19) and breakdown of redox enzyme [Fe-S] clusters (20), and cell membrane dysfunction (11), were explored to have a general view of the various diphenylurea compounds’ mechanism of action.

A well-established method to evaluate bacteria viability, propidium iodide (PI) staining, was performed to assess the membrane permeability. Compounds **59** and **61** showed no effect on the cell membrane of Gram-negative bacteria, *P. aeruginosa*. However, both compounds were effective at cell membrane disruption of *MRSA* strains, proving the effective bactericidal activity of such compounds on the Gram-positive bacteria’s cell membrane (Fig. S2). Further evaluations, including reduced thiol, iron detection Ferine-S, and hydrogen peroxide levels, were performed to explore other possible mechanisms of action of these compounds. These assays demonstrated that none of these novel compounds have any effect on these processes of toxicity.

Due to the considerable structural differences between the Gram-positive and Gram-negative cell walls of bacteria (21), as well as eukaryotic cells (22), certain antibiotics, such as polycations and chelators, selectively target the distinctive structure of bacterial cell walls (23). A thin layer of peptidoglycan and a thick layer of lipopolysaccharide and lipoprotein-containing outer membrane compose the cell wall of Gram-negative bacteria. This is important because it regulates which molecules enter the cell and even which ones are expelled from it. As a result, the susceptibilities of Gram-positive and Gram-negative bacteria to antimicrobial drugs differ due to their different cell wall structures. This could account for the abovementioned chemicals’ effectiveness against Gram-positive bacteria but not against Gram-negative bacteria (24). This targeted approach allows antibiotics to eradicate bacteria more specifically while preserving host cells (23). In our investigation of membrane disruption potency within our chosen components, we employed the membrane leakage probe (PI), which binds to DNA, to assess membrane destabilization following a 1-hour exposure to our agents. The results indicate that all selected agents disrupted the Gram-positive cell membrane compared with the untreated group (*P* < 0.001), but not the sig effect of the Gram-negative cell membrane. This indicates possibly originating from severe membrane deformations, which is an indication that the primary antibacterial mode of action of these compounds may involve cell membrane damage (5).

### Toxicity in eukaryotic cell lines

We selected compounds EPB-59, EPB-61, and EPB-62 due to their elevated antibacterial activity. Subsequently, we evaluated their cytotoxicity in eukaryotic cell lines derived from human, canine, or simian origins. In HEL, HeLa, MT4, and VERO cells, the minimum cytotoxic concentration (MCC) or CC_50_ values were similar for the three compounds and in the order of 4–20 µM. EPB-59 and EPB-61 showed lower cytotoxicity in canine Madin-Darby canine kidney (MDCK) cells, demonstrating a CC_50_ value ≥100 µM (Table 3). The CC50 values of EPB-59 and EPB-61 on MDCK cells reveal their exceptional cytotoxic profiles in comparison to other compounds, with concentrations varying from ≤0.8 µM to a > 100 µM. Notably, EPB-59 and EPB-61 possess cytotoxicity levels 2 logs lower than that of EPB-34.

**TABLE 3:** Eukaryotic cell toxicity of different diphenyl-urea analogs

	Cell line (*in vitro* cytotoxicity, µM)				
	Human			Mouse	Monkey
	HEL^*a*^ (*MCC*)^*b*^	HeLa^*c*^ (*MCC*)^*b*^	MT4^*d*^ (*MCC*)^*b*^	MDCK^*e*^ (*CC_50_*)^*f*^	Vero^*g*^ (*CC_50_*)^*f*^
EBP-59	>4	4	3.9	>100	>4
EBP-61	>4	4	2.8	>100	>4
EBP-62	20	>4	4.7	100	>4

^*a*^ HEL: human leukemia cells.

^*b*^ MCC: minimum compound concentration that causes a microscopically detectable alteration of normal cell morphology.

^*c*^ HeLa: human cervical cancer cells.

^*d*^ MT4: human T leukemia cells.

^*e*^ MDCK: Madin-Darby canine kidney cells.

^*f*^ 50% Cytotoxic concentration, as determined by measuring the cell viability with the colorimetric formazan-based MTS assay. Values shown are the mean of 2 or 3 determinations.

^*g*^ Vero: Green monkey kidney cells.

Consistent with these findings, Dr. Manuel’s group previously (5, 11) addressed cytotoxicity issues related to new compounds with replacements for pentafluorosulfanyl groups. At first, substituting this group for a chlorine atom appeared promising, but high cytotoxicity in certain derivatives presented difficulties. We looked into taking an atom of chlorine out of these compounds in order to solve this. The modified ureas 7 (#**62** in this study) and 8 (#**61** in this study) showed promise by having cytotoxicity and antibacterial activity comparable with TCC; this is confirmed in this study as well. Compound 9 (#**55** in this study) was created by substituting a second pentafluorosulfanyl group for the remaining chlorine atom in compound 7, which showed similar cytotoxicity to TCC but a wider range of activity. Comparably, switching from compound 8 to compound 10 (#**33** in this study) resulted in a very promising chemical that had a selectivity index as high as 412 and was effective against five strains of Gram-positive bacteria. The selectivity index (SI) serves as a valuable indicator for drug safety, considering both therapeutic efficacy and toxicity. A higher SI value indicates that a compound is less cytotoxic to normal cells and potentially safer. Furthermore, adding a fourth electron-withdrawing group to cloflucarban for a brief period of time has been investigated. Compounds 12–14 (#**34**, **37**, and **56**, respectively, in this study) showed activity against five strains of Gram-positive bacteria with cytotoxicities that were comparable with or slightly higher compared with those of TCC and cloflucarban. Our analysis indicates that there might not be many benefits to adding a fourth electron-withdrawing group. In another study (25), three compounds were assessed in a different investigation on the toxicity of mammalian cells, and they showed exceptional safety profiles against human colorectal cells (Caco-2). Pentafluorosulfanyl 13 (#**37** in this study) substance was found to be non-toxic to Caco-2 cells at concentrations up to 64 µg mL^−1^, which is 128 times over its MIC against *MRSA* ATCC 33592. This finding reinforces the molecule’s potential for use in clinical settings.

### *In vitro* results

The new diphenylurea compounds studied here were not effective against Gram-negative pathogens such as *E. coli, P. aeruginosa, Klebsiella pneumoniae,* and *P. mirabilis* except compound **59**, which had acceptable potency regarding MIC, MBC, and MBIC to *P. mirabilis* spp. This could indicate that variations in diphenyl-urea antibiotics could make them efficient to use against Gram-negative bacteria. Perhaps, this compound and how it differs from others could be a future topic for further study. Furthermore, promising results regarding the application of compounds **59**, **61**, and **62** were achieved with Gram-positive bacteria such as *S. aureus, S. epidermidis,* and *MRSA* specifically with compound **59**, having relatively superior results while factoring cell cytotoxicity in all cell lines evaluated. Although results regarding the use of such compounds on *MRSA* species did not accompany a safe dose regarding human cell lines, MIC, MBC, and MBIC measurements of 10 *MRSA* clinical isolates showed that these compounds could be used in a rather safe measure when evaluated on clinical isolates. All *MRSA* clinical isolates showed susceptibility to compounds **59**, **61**, and **62** regarding MBIC and MIC (Table 2). With compound **59** yet again proving to be the best option across all measurements regarding safety and efficacy, all these compounds showed a significantly higher potency when compared with conventional antibiotics evaluated.

Other compounds were also evaluated in this study. However, many of them did not prove as effective as the three discussed above. Compound **34** showed promising results regarding MIC in Gram-positive bacteria assessed in this study with a similar cytotoxicity profile as compound **59** regarding HeLa, HEL, and Vero cell lines. Unfortunately, the same does not apply in the context of MBIC and MBC. Compound **37** showed slightly worse results, and its cytotoxicity was not assessed. Compound **40** did not show any promising effectiveness against Gram-positive bacteria even in comparison to conventional antibiotics.

### *In vivo* efficacy

**Fig 2:**
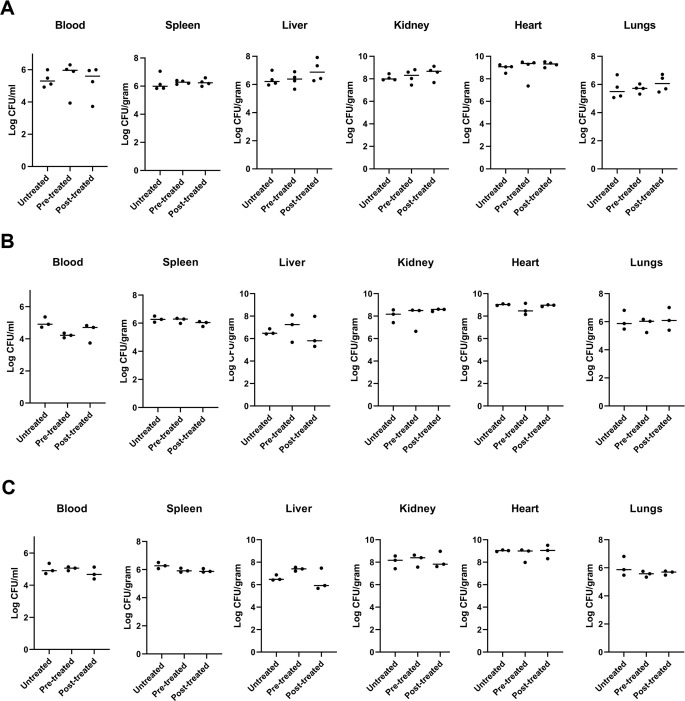
Intravenous *MRSA* infection model to screen for *in vivo* effectivity of compounds **59, 61,** and **62**. CFU enumerations for blood, spleen, liver, kidney (left), heart, and lungs after 24 hours of infection with *S. aureus* MW2. Mice were left untreated or were treated 1 hour before (pre-treated) or after infection (post-treated) with compound **59** (**A**), **61** (**B**), or **62** (**C**). One-way ANOVA with Bonferroni’s post-test, data not significant.

The most effective compounds, **59**, **61**, and **62**, were selected to evaluate their *in vivo* efficacy. Mice were infected systemically with the community-associated *MRSA* strain MW2 at an infectious dose of 5 × 10^7^ colony-forming units (CFUs) as described (26). Treatment groups were injected intraperitoneally with the compound of interest 1 hour before or after infection, with a saline-injected control group. After 24 hours of infection, mice were sacrificed, and blood, peritoneal fluid, spleen, liver, kidney (left), heart, and lungs were harvested for CFU enumeration to determine the bacterial burden in these organs. These experiments revealed a consistently high bacterial burden in all organs of the animals 24 hours after treatment, irrespective of the specific treatment administered (Fig. 2). The single-dose treatment failed to reduce bacterial counts in any of the animal organs, even after experimenting with different compound concentrations. Although disappointing, the lack of promising results in the single-dose treatments may have a variety of reasons, including rapid metabolism and excretion of the compounds by the liver, or the acknowledged intracellular aspect of *S. aureus* in its infectious cycle (27). Consequently, further research is imperative to determine the *in vivo* efficacy of compounds **59**, **61**, and **62**, specifically if a pulsatile and continuous treatment over 2–4 days may prove to be more effective. Additionally, investigating the possibility of topical application for these components presents another viable option.

### Conclusion

Overall, for the first evaluation of such compounds, the results demonstrated Gram class selectivity and cell membrane-associated mechanism of antimicrobial activity. Cell toxicity and animal results indicate challenges but still suggest that there is promise to explore applications of these novel compounds as useful antimicrobials moving forward.

## MATERIALS AND METHODS

### Pentafluorosulfanyl-containing triclocarban analogs preparation and characterization

All the pentafluorosulfanyl-containing diarylureas were synthesized as previously reported (5, 11, 12). In all cases, the analytical and spectroscopic data matched with those previously reported in the abovementioned three references.

### Bacterial preparation

All bacterial strains subjected to testing had been stored at −70°C prior to the experiment. The experiment commenced by streaking small amounts of the frozen bacterial samples onto Muller-Hinton agar (MHA) culture medium (BD Bacto, Oxoid, Basingstoke, UK Cat# X296B) and allowing for overnight incubation. Bacteria were re-cultured on MHA plates as required to confirm colony morphology and lack of contamination. Strains tested were *P. aeruginosa* ATCC 27853, *E. coli* ATCC 25922, *Staphylococcus aureus* ATCC 25923, *MRSA* ATCC 33591, *S. epidermidis* ATCC 12228, *Klebsiella pneumoniae* ATCC, *P. mirabilis* ATCC 35659, and 10 clinical isolates of *MRSA* from Foothills Hospital, Calgary, Canada. To prepare for susceptibility testing, a protocol modified from Lemire et al was used (28). MHB was swab-inoculated from MHA colonies and shake-incubated until turbid. Dilution of the broth with 0.9% saline was performed as necessary to match a 1.0 McFarland standard.

### Planktonic susceptibility

MIC and MBC assessments were conducted on all bacterial strains using 96-well microtiter plates. To prepare the wells for bacteria, serial dilutions of the antibacterial agents were carried out along the rows of two microtiter plates. The antibacterials were 2-fold diluted with Mueller-Hinton broth (MHB) in each column, reaching a volume of 75 µL. The 1.0 McFarland standard was diluted 15-fold in MHB, and 75 µL of inoculum was added to the wells treated with antibacterials, resulting in a total volume of 150 µL. The microtiter plates were covered and then shake-incubated overnight at 150 rpm and 37°C. MIC was determined based on visible bacterial growth. In cases of antibacterial opacity or ambiguous results, streaking of well cultures on MHA, overnight incubation, and subsequent comparison with controls were performed. MBC was determined by transferring 3 µL of culture from all MIC microtiter plate wells to 147 µL of Mueller-Hinton broth in fresh plates. These plates were shake-incubated overnight at 150 rpm and 37°C, and bacterial growth was visually inspected.

### Inhibition of biofilm formation

Minimum biofilm inhibitory concentration (MBIC) determination coincided with MIC and MBC assessments. The Calgary Biofilm Device (CBD) was employed to cover the 150 µL wells of the MIC microtiter plate. Following overnight incubation, the MIC plates and pegs underwent three washes with 250 µL of distilled water (dH_2_O) to eliminate planktonic bacteria. Biofilms were then stained with 200 µL of a 0.1% crystal violet solution for 30 minutes. Post-staining, microplates and pegs were washed three times with 200 µL of ddH_2_O to remove excess dye. Biofilm quantification involved sonication using a 250HT ultrasonic cleaner (VWR International), set at 60 Hz for 10 minutes, into 200 µL of 70% ethanol. Absorbance readings at 600 nm were taken, with 70% ethanol serving as the blank (1).

### Membrane permeability measurements

For assessing membrane permeability, PI (Invitrogen, Eugene, Oregon, USA) served as the fluorescent reporter dye. Increased PI fluorescence readings are indicative of heightened membrane disruption and permeability, as PI can penetrate cells, bind to DNA, and remain within the cells (29, 30). Bacteria were cultured in 3 mL of Mueller-Hinton broth (MHB) and incubated at 37°C for approximately 3 hours in a shaker incubator (150 rpm) until reaching an OD600 (optical density) of 0.08. Each group received treatment with MIC concentrations of agents, the untreated groups with phosphate-buffered saline (PBS) as a negative control, and a positive control involving bacteria subjected to freezing and boiling three times (frozen for 5 minutes at −80°C and incubated at 90°C for 10 minutes) to disrupt the bacterial cell membrane. All treated and control groups were incubated at 37°C for 1 hour in a shaker incubator (150 rpm). Ciprofloxacin and gentamycin were employed as internal controls, as they are known not to target the bacterial cell membrane in the initial steps. After incubation, the samples were centrifuged, washed with PBS (10,000 × *g* for 2 minutes), and bacteria were stained with 0.08 mM PI for 5 minutes at 21°C in the dark. Subsequently, 10 µL of samples were transferred onto slides and examined under a fluorescence microscope (Zeiss Axio Imager Z1) using the same exposure time (640 ms red and 1 s green). Densitometry analysis was conducted using Fiji software (ImageJ).

### Hydrogen peroxide assay

The hydrogen peroxide concentration after exposure to our agents was detected with the Pierce Quantitative Peroxide Assay Kit in the aqueous-compatible formulation according to the manufacturer’s instructions (31). For preparing the standard, 1 mM solution of H_2_O_2_ was initially made by diluting a 30% H_2_O_2_ stock 1:9000 (11 µL of 30% H_2_O_2_ into 100 mL of double-distilled (DD) water). This sample was then serially diluted with DD water 1:2 (100 µL of DD water + 100 µL of the previous dilution) for a total of 11 samples as a standard. Two hundred microliters of the working reagent (WR) from the kit were added to 20 µL of the diluted H_2_O_2_ standards. Samples were mixed and incubated for 15 minutes at 21°C in the dark. Absorbances were measured at 595 nm using a Thermomax microtiter plate reader with Softmax Pro data analysis software (Molecular Devices, Sunnyvale, CA).

For measuring the treated and untreated samples, the bacteria were cultured in 2 mL of MHB and were incubated at 37°C for ~3 hours in a shaker incubator (150 rpm) to reach the OD600 of 0.08. Then, they were treated with MIC concentrations of agents and untreated groups with PBS as a negative control; the positive control was treated with 250 µM H_2_O_2_ and incubated at 37°C for 1 hour in a shaker incubator (150 rpm). Ciprofloxacin and gentamycin were used as antibiotic comparators. The bacterial cells were washed with PBS by centrifuging (10,000 × *g* for 5 minutes), and the supernatant was then discarded. Two milliliters of PBS were added to each sample and vortexed, and 200 µL of the WR was added to 20 µL of each sample. Samples were mixed and incubated for 15 minutes at room temperature. Absorbances were measured at 595 nm using a Thermomax microtiter plate reader with Softmax Pro data analysis software (Molecular Devices, Sunnyvale, CA). The blank value was subtracted from all sample measurements. The samples’ H_2_O_2_ concentrations were calculated based on standard carve R^2^ = 0.93 value.

### Iron detection Ferene-S assay

The release of Fe^2+^ from the iron-sulfur clusters in *P. aeruginosa* and *MRSA* was detected using a Ferene-S assay with the probe, 3-(2pyridyl)−5,6-bis(2-(5-fury lsulfonic acid))−1,2,4-triazin (Sigma-Aldrich, St Louis, MO, USA) (32). Ten milliliters of bacteria (OD600 of 0.08) were prepared in Tris-HCl buffer (20 mM, pH 7). The bacterial cells were washed with the same buffer by centrifuging (10,000 × *g* for 5 minutes), and the supernatant was discarded. The platelet (bacterial cells) was then lysed by sonication using a 250HT ultrasonic cleaner (VWR International), set at 60 Hz for 20 minutes in the same buffer. The samples were centrifuged (10,000 × *g* for 5 minutes), and the supernatant was collected. The solution was treated with MIC concertation of agents, untreated control (PBS), and positive control (incubated at 90°C for 10 minutes to break down the Fe-sulfur cluster). Then, a 10 mM Ferene-S probe was added to each sample in a 96-well plate, and samples were incubated at 21°C in dark for 1 hour. Absorbance was measured at 600 nm, using a Thermomax microtiter plate reader with Softmax Pro data analysis software (Molecular Devices, Sunnyvale, CA) (29).

### Reduced thiol (RSH) assay

The accuracy of the assay was assessed with a standard dilution of reduced glutathione (≥98%, Alfa Aesar, Germany) and oxidized glutathione (Sigma, USA). For preparing the standard, 1 mM solution of each glutathione was serially diluted with 50 mM Tris/HCl pH 81:2 (150 µL of 50 mM Tris/HCl pH 8 + 150 µL of glutathione) for a total of 11 samples as a standard. Then, 0.1 mM of Ellman’s reagent 5,5’-dithiobis(2-nitrobenzoic acid) (DTNB) was added to each well. Samples were mixed and incubated for 30 minutes at 37°C in dark. Absorbances were measured at 412 nm using a Thermomax microtiter plate reader with Softmax Pro data analysis software (Molecular Devices, Sunnyvale, CA).

For measuring the treated and untreated samples with agents, the bacteria were cultured in 3 mL of MHB and were incubated at 37°C for ~3 hours in a shaker incubator (150 rpm) to reach the OD600 of 0.08. Then, they were treated with MIC concentrations of agents and untreated groups with PBS (as a negative control) and incubated at 37°C for 1 hour in a shaker incubator (150 rpm). The bacterial cells were washed with PBS by centrifuging (10,000 × *g* for 5 minutes), and the supernatant was discarded. One milliliter of 50 mM Tris/HCl pH 8.0, 5 mM EDTA, 0.1% SDS, and 0.1 mM DTNB was added to each sample and vortexed. These cell suspensions were incubated at 37°C for 30 minutes and then centrifuged in a microfuge for 1 minute at 15,000 g. Absorbances were measured at 412 nm using a Thermomax microtiter plate reader with Softmax Pro data analysis software (Molecular Devices, Sunnyvale, CA). The blank value was subtracted from all sample measurements. The absorption coefficient of oxidized DTNB (1.36 × 10^4^ M^−1^ cm^−1^) at this wavelength was then used to calculate the RSH concentration of the cell.

### Eukaryotic cell toxicity of pentafluorosulfanyl-containing triclocarban compounds

Five eukaryotic cell lines were used to determine the cytotoxicity of the compounds: human embryonic lung (HEL) fibroblast cells, human cervixcarinoma-derived HeLa cells, human T-cell leukemia-derived MT4 cells, MDCK cells, and African Green monkey kidney-derived VERO cells. Semi-confluent cell cultures in 96-well plates were exposed to serial dilutions of the compounds or to medium (= no compound control), then incubated at 37°C. Four days later, the cells were inspected by microscopy to determine the MCC, that is, compound concentration that causes a microscopically detectable alteration of normal cell morphology. Next, the MTS cell viability reagent (CellTiter 96 AQueous MTS Reagent from Promega) was added. After 4 hours of incubation at 37°C, OD at 490 nm was recorded in a microplate reader. The percentage cytotoxicity was calculated as: [1 − (OD_Cpd_)/(OD_Contr_)] × 100, after which the 50% cytotoxicity value (CC_50_) was derived by extrapolation, assuming semi-log dose response.

### Bacterial infection and mouse treatment

Animal experiments were performed with 6- to 10-week/old adult male mice. Mice were kept in a pathogen-free facility under standardized conditions: temperature of 21–22°C, illumination of 12-hour light–12-hour dark, and access to tap water and food. Experimental animal protocols were approved by the University of Calgary Animal Care Committee and followed the Canadian Council for Animal Care Guidelines.

MW2 *Staphylococcus aureus* was grown on brain heart infusion (BHI) agar plates (BD biosciences). A single colony was grown overnight in BHI medium (BD biosciences) at 37°C while shaking. Subcultures of 100 mL of the overnight (ON) culture were grown in 3 mL of the same culture medium for 2 hours at 37°C while shaking to reach the exponential growth phase. Bacteria were brought to a concentration of OD_660_ 1.0 in saline. A 1:4 dilution of bacteria was prepared, and animals were infected through an intravenous catheter with 200 mL of the bacterial suspension so that bacterial dose was controlled to a dose of 5 × 10^7^ bacteria. Mice were treated with an intraperitoneal injection of 100 µl of a compound of interest (5 mM) 1 hour before or after infection. The untreated group was injected 1 hour post-infection with 100 µl saline.

For assessing bacterial by CFU enumeration, the mice were anesthetized with isoflurane (Fresenius Kabi) and washed with 70% ethanol to collect blood and sacrificed to further collect liver, spleen, kidney (left), heart, and lungs. Blood was collected by cardiac puncture and pooled with 40 mL heparin. Twenty-five milliliters of 1:10 diluted blood samples were plated. Organ samples were weighed and homogenized in 1 mL PBS and serially diluted (spleen, liver, and lung samples 1:10, 1:100; 1:1000; and 1:10 000; kidney and heart samples 1:100; 1:1000; 1:10 000; and 1:100 000). Thirty milliliters of each dilution were plated at a 30° angle to form stripes. All plates were incubated ON at 37°C. Cultures were counted, and CFUs were calculated taking the weight of the organ into account.

For statistical analysis, one-way ANOVA with Bonferroni’s post-test was used in this study. All data are from a minimum of three biological replicates.

## SUPPLEMENTAL MATERIAL

Supplemental material spectrum.00071-24-s0001.docx Fig. S1 and S2.

ASM does not own the copyrights to Supplemental Material that may be linked to, or accessed through, an article. The authors have granted ASM a non-exclusive, world-wide license to publish the Supplemental Material files. Please contact the corresponding author directly for reuse.

## References

[1] Getahun H, Smith I, Trivedi K, Paulin S, Balkhy HH. 2020. Tackling antimicrobial resistance in the COVID-19 pandemic. Bull World Health Organ 98:442. doi:10.2471/BLT.20.268573 32742026 PMC7375214

[2] Pushpakom S, Iorio F, Eyers PA, Escott KJ, Hopper S, Wells A, Doig A, Guilliams T, Latimer J, McNamee C, Norris A, Sanseau P, Cavalla D, Pirmohamed M. 2019. Drug repurposing: progress, challenges and recommendations. Nat Rev Drug Discov 18:41–58. doi:10.1038/nrd.2018.168 30310233

[3] Catalano A, Iacopetta D, Pellegrino M, Aquaro S, Franchini C, Sinicropi MS. 2021. Diarylureas: repositioning from antitumor to antimicrobials or multi-target agents against new pandemics. Antibiotics (Basel) 10:92. doi:10.3390/antibiotics10010092 33477901 PMC7833385

[4] Farha MA, Brown ED. 2019. Drug repurposing for antimicrobial discovery. Nat Microbiol 4:565–577. doi:10.1038/s41564-019-0357-1 30833727

[5] Pujol E, Blanco-Cabra N, Julián E, Leiva R, Torrents E, Vázquez S. 2018. Pentafluorosulfanyl-containing triclocarban analogs with potent antimicrobial activity. Molecules 23:2853. doi:10.3390/molecules23112853 30400165 PMC6278391

[6] Altomonte S, Zanda M. 2012. Synthetic chemistry and biological activity of pentafluorosulphanyl (SF5) organic molecules. J Fluor Chem 143:57–93. doi:10.1016/j.jfluchem.2012.06.030

[7] Savoie PR, Welch JT. 2015. Preparation and utility of organic pentafluorosulfanyl-containing compounds. Chem Rev 115:1130–1190. doi:10.1021/cr500336u 25341449

[8] Bassetto M, Ferla S, Pertusati F. 2015. Polyfluorinated groups in medicinal chemistry. Future Med Chem 7:527–546. doi:10.4155/fmc.15.5 25875877

[9] Gujjar R, El Mazouni F, White KL, White J, Creason S, Shackleford DM, Deng X, Charman WN, Bathurst I, Burrows J, Floyd DM, Matthews D, Buckner FS, Charman SA, Phillips MA, Rathod PK. 2011. Lead optimization of aryl and aralkyl amine-based triazolopyrimidine inhibitors of Plasmodium falciparum dihydroorotate dehydrogenase with antimalarial activity in mice. J Med Chem 54:3935–3949. doi:10.1021/jm200265b 21517059 PMC3124361

[10] Moraski GC, Bristol R, Seeger N, Boshoff HI, Tsang P-Y, Miller MJ. 2017. Preparation and evaluation of potent pentafluorosulfanyl-substituted anti-tuberculosis compounds. ChemMedChem 12:1108–1115. doi:10.1002/cmdc.201700170 28654200 PMC5603227

[11] Probst A, Pujol E, Häberli C, Keiser J, Vázquez S. 2021. In vitro, in vivo, and absorption, distribution, metabolism, and excretion evaluation of SF5-containing N, N’-diarylureas as antischistosomal agents. Antimicrob Agents Chemother (Bethesda) 65:00615–00621. doi:10.1128/aac.00615-21 PMC8451275 34310210

[12] Zarei M, Pujol E, Quesada-López T, Villarroya F, Barroso E, Vázquez S, Pizarro-Delgado J, Palomer X, Vázquez-Carrera M. 2019. Oral administration of a new HRI activator as a new strategy to improve high‐fat‐diet‐induced glucose intolerance, hepatic steatosis, and hypertriglyceridaemia through FGF21. Br J Pharmacol 176:2292–2305. doi:10.1111/bph.14678 30927369 PMC6555855

[13] Chen T, Ozel D, Qiao Y, Harbinski F, Chen L, Denoyelle S, He X, Zvereva N, Supko JG, Chorev M, Halperin JA, Aktas BH. 2011. Chemical genetics identify eIF2α kinase heme-regulated inhibitor as an anticancer target. Nat Chem Biol 7:610–616. doi:10.1038/nchembio.613 21765405 PMC3684262

[14] Garuti L, Roberti M, Bottegoni G, Ferraro M. 2016. Diaryl urea: a privileged structure in anticancer agents. Curr Med Chem 23:1528–1548. doi:10.2174/0929867323666160411142532 27063259

[15] Walsh SE, Maillard J-Y, Russell AD, Catrenich CE, Charbonneau DL, Bartolo RG. 2003. Activity and mechanisms of action of selected biocidal agents on Gram‐positive and‐negative bacteria. J Appl Microbiol 94:240–247. doi:10.1046/j.1365-2672.2003.01825.x 12534815

[16] Xie Y, Wang L, Yang Y, Zha L, Zhang J, Rong K, Tang W, Zhang J. 2022. Antibacterial and anti-biofilm activity of diarylureas against Enterococcus faecium by suppressing the gene expression of peptidoglycan hydrolases and adherence. Front Microbiol 13:1071255. doi:10.3389/fmicb.2022.1071255 36590419 PMC9797508

[17] Wu H, Moser C, Wang HZ, Høiby N, Song ZJ. 2015. Strategies for combating bacterial biofilm infections. Int J Oral Sci 7:1–7. doi:10.1038/ijos.2014.65 25504208 PMC4817533

[18] Ceri H, Olson ME, Stremick C, Read RR, Morck D, Buret A. 1999. The Calgary Biofilm Device: new technology for rapid determination of antibiotic susceptibilities of bacterial biofilms. J Clin Microbiol 37:1771–1776. doi:10.1128/JCM.37.6.1771-1776.1999 10325322 PMC84946

[19] Reimche JL, Kirse DJ, Whigham AS, Swords WE. 2017. Resistance of non-typeable Haemophilus influenzae biofilms is independent of biofilm size. Pathog Dis 75:ftw112. doi:10.1093/femspd/ftw112 27956464 PMC5353992

[20] Wannigama DL, Hurst C, Hongsing P, Pearson L, Saethang T, Chantaravisoot N, Singkham-In U, Luk-In S, Storer RJ, Chatsuwan T. 2020. A rapid and simple method for routine determination of antibiotic sensitivity to biofilm populations of Pseudomonas aeruginosa. Ann Clin Microbiol Antimicrob 19:8. doi:10.1186/s12941-020-00350-6 32169075 PMC7071750

[21] Pinto N de C, Campos LM, Evangelista ACS, Lemos ASO, Silva TP, Melo RCN, de Lourenço CC, Salvador MJ, Apolônio ACM, Scio E, Fabri RL. 2017. Antimicrobial Annona muricata L.(soursop) extract targets the cell membranes of Gram-positive and Gram-negative bacteria. Industrial Crops and Products 107:332–340. doi:10.1016/j.indcrop.2017.05.054

[22] Theriot JA. 2013. Why are bacteria different from eukaryotes? BMC Biol 11:1–17. doi:10.1186/1741-7007-11-119 24330667 PMC3874686

[23] Vaara M. 1992. Agents that increase the permeability of the outer membrane. Microbiol Rev 56:395–411. doi:10.1128/mr.56.3.395-411.1992 1406489 PMC372877

[24] Silhavy TJ, Kahne D, Walker S. 2010. The bacterial cell envelope. Cold Spring Harb Perspect Biol 2:a000414. doi:10.1101/cshperspect.a000414 20452953 PMC2857177

[25] Naclerio GA, Abutaleb NS, Onyedibe KI, Seleem MN, Sintim HO. 2020. Potent trifluoromethoxy, trifluoromethylsulfonyl, trifluoromethylthio and pentafluorosulfanyl containing (1, 3, 4-oxadiazol-2-yl) benzamides against drug-resistant Gram-positive bacteria. RSC Med Chem 11:102–110. doi:10.1039/c9md00391f 33479609 PMC7536820

[26] Surewaard BGJ, Deniset JF, Zemp FJ, Amrein M, Otto M, Conly J, Omri A, Yates RM, Kubes P. 2016. Identification and treatment of the Staphylococcus aureus reservoir in vivo. J Exp Med 213:1141–1151. doi:10.1084/jem.20160334 27325887 PMC4925027

[27] Hommes JW, Surewaard BGJ. 2022. Intracellular habitation of Staphylococcus aureus: molecular mechanisms and prospects for antimicrobial therapy. Biomedicines 10:1804. doi:10.3390/biomedicines10081804 36009351 PMC9405036

[28] Lemire JA, Kalan L, Gugala N, Bradu A, Turner RJ. 2017. Silver oxynitrate–an efficacious compound for the prevention and eradication of dual-species biofilms. Biofouling 33:460–469. doi:10.1080/08927014.2017.1322586 28521545

[29] Morones-Ramirez JR, Winkler JA, Spina CS, Collins JJ. 2013. Silver enhances antibiotic activity against Gram-negative bacteria. Sci Transl Med 5:190ra81. doi:10.1126/scitranslmed.3006276 PMC3771099 23785037

[30] Novo DJ, Perlmutter NG, Hunt RH, Shapiro HM. 2000. Multiparameter flow cytometric analysis of antibiotic effects on membrane potential, membrane permeability, and bacterial counts of Staphylococcus aureus and Micrococcus luteus. Antimicrob Agents Chemother 44:827–834. doi:10.1128/AAC.44.4.827-834.2000 10722477 PMC89778

[31] Thermo Fisher Scientific 2012. Pierce Quantitative Peroxide Assay Kits. Available from: https://www.thermofisher.com/document-connect/document-connect.html?url=https%3A%2F%2Fassets.thermofisher.com%2FTFS-Assets%2FLSG%2Fmanuals%2FMAN0011275_Pierce_Quant_Peroxide_Asy_UG.pdf. Retrieved May 2023.

[32] Hennessy DJ, Reid GR, Smith FE, Thompson SL. 1984. Ferene—a new spectrophotometric reagent for iron. Can J Chem 62:721–724. doi:10.1139/v84-121

