# Supplementary material for: Novel pentafluorosulfanyl-containing triclocarban analogs selectively kill Gram-positive bacteria
Source: Microbiol Spectr. 2024 May 3;12(6):e00071-24. doi: 10.1128/spectrum.00071-24 (PMC11237694; doi:10.1128/spectrum.00071-24)
Supplement: Supplemental material — Fig. S1 and S2. [file spectrum.00071-24-s0001.docx]

***Supplementary***

**Novel** **Pentafluorosulfanyl-containing Triclocarban Analogs selectively kill Gram-positive bacteria.**

Ali Pormohammad^1,2^, Melika Moradi^1,3^, Josefien W. Hommes^4^, Eugènia Pujol^5,6^, Lieve Naesens^7^, Santiago Vázquez^5,6^, Bas G. J. Surewaard^4^, Mohammad Zarei^8,9^, Manuel Vazquez-Carrera^6,10,11,12^*, Raymond J. Turner^1^*

***** Corresponding authors:

Raymond J. Turner: Mail address: 447-Biological Sciences Building, 2500 University Dr. NW, Calgary, Alberta T2N 1N4, Canada; Tel.: +1-403-220-4308 [turnerr@ucalgary.ca](mailto:turnerr@ucalgary.ca)

Manuel Vazquez-Carrera: Mail address: Unitat de Farmacologia. Facultat de Farmàcia i Ciències de l’Alimentació. Av. Joan XXIII 27-31. Barcelona E-08028. Spain. Tel: +34 934024531. Email: mvazquezcarrera@ub.edu

| ***MRSA* clinical isolates** | | | | ***MRSA* #1** | ***MRSA* #2** | ***MRSA* #3** | ***MRSA* #4** | ***MRSA* #5** | ***MRSA* #6** | ***MRSA* #7** | ***MRSA* #8** | ***MRSA* #9** | **MRSA #10** |
| --- | --- | --- | --- | --- | --- | --- | --- | --- | --- | --- | --- | --- | --- |
| **Planktonic** | **MIC** (mM) | diphenyl-urea | **EBP-34** | 0.6 | 0.001 | 0.08 | 0.001 | 0.01 | 0.01 | 0.001 | 0.005 | 0.001 | 0.001 |
|  |  |  | **EBP-37** | 0.01 | 0.001 | 0.08 | 0.001 | 0.01 | 0.001 | 0.01 | 0.001 | 0.005 | 0.001 |
|  |  |  | **EBP-40** | 0.02 | 0.02 | 0.08 | 0.04 | 0.02 | 0.04 | 0.04 | 0.04 | 0.02 | 0.04 |
|  |  |  | **EBP-59** | 0.001 | 0.001 | 0.001 | 0.001 | 0.001 | 0.001 | 0.001 | 0.001 | 0.001 | 0.001 |
|  |  |  | **EPB-61** | 0.001 | 0.001 | 0.001 | 0.001 | 0.001 | 0.001 | 0.001 | 0.001 | 0.001 | 0.001 |
|  |  |  | **EBP-62** | 0.001 | 0.001 | 0.001 | 0.001 | 0.001 | 0.001 | 0.005 | 0.005 | 0.001 | 0.005 |
|  |  | Antibiotics | **Cip** | 0.4 | 0.05 | 0.4 | 0.05 | 0.2 | 0.2 | 0.2 | 1.6 | 0.4 | 0.05 |
|  |  |  | **Gen** | 0.1 | 0.05 | 0.05 | 0.05 | 0.8 | 3 | 0.05 | 0.05 | 0.05 | 0.05 |
|  | **MBC** (mM) | diphenyl-urea | **EBP-34** | 0.001 | 0.001 | 0.08 | 0.001 | 0.01 | 0.01 | 0.01 | 0.005 | 0.001 | 0.01 |
|  |  |  | **EBP-37** | 1.2 | 0.01 | 0.6 | 0.01 | 0.01 | 0.001 | 0.04 | 0.01 | 0.01 | 0.01 |
|  |  |  | **EBP-40** | 1.2 | 0.04 | 0.16 | 0.16 | 0.04 | 0.16 | 0.08 | 0.08 | 0.08 | 0.01 |
|  |  |  | **EBP-59** | 0.005 | 0.001 | 0.001 | 0.005 | 0.01 | 0.001 | 0.001 | 0.005 | 0.02 | 0.04 |
|  |  |  | **EPB-61** | 0.04 | 0.01 | 0.01 | 0.02 | 0.04 | 0.02 | 0.02 | 0.04 | 0.04 | 0.02 |
|  |  |  | **EBP-62** | 0.08 | 0.005 | 0.001 | 0.02 | 0.02 | 0.005 | 0.01 | 0.08 | 0.04 | 0.08 |
|  |  | Antibiotics | **Cip** | 1.6 | 0.4 | 0.05 | 0.4 | 0.4 | 0.4 | 0.05 | 12.5 | 1.6 | 0.8 |
|  |  |  | **Gen** | 1.6 | 0.2 | 0.05 | 0.05 | 6.25 | 25 | 1.6 | 0.05 | 0.05 | 0.05 |
| **Biofilm** | **MBIC** (mM) | diphenyl-urea | **EBP-34** | 1.2 | 0.001 | 0.08 | 0.001 | 0.01 | 0.01 | 0.02 | 0.005 | 0.001 | 0.001 |
|  |  |  | **EBP-37** | 0.02 | 0.001 | 0.3 | 0.01 | 0.01 | 0.001 | 0.01 | 0.001 | 0.001 | 0.001 |
|  |  |  | **EBP-40** | 0.001 | 0.001 | 0.08 | 0.001 | 0.02 | 0.04 | 0.001 | 0.001 | 0.001 | 0.001 |
|  |  |  | **EBP-59** | 0.001 | 0.001 | 0.001 | 0.001 | 0.001 | 0.001 | 0.001 | 0.001 | 0.001 | 0.001 |
|  |  |  | **EPB-61** | 0.001 | 0.001 | 0.001 | 0.001 | 0.001 | 0.001 | 0.001 | 0.001 | 0.001 | 0.001 |
|  |  |  | **EBP-62** | 0.001 | 0.001 | 0.001 | 0.001 | 0.001 | 0.001 | 0.001 | 0.001 | 0.001 | 0.001 |
|  |  | Antibiotics | **Cip** | 0.05 | 0.05 | 0.2 | 0.05 | 0.05 | 0.2 | 0.05 | 0.05 | 0.05 | 0.05 |
|  |  |  | **Gen** | 0.05 | 0.05 | 0.05 | 0.05 | 0.1 | 12.5 | 0.05 | 0.05 | 0.05 | 0.05 |


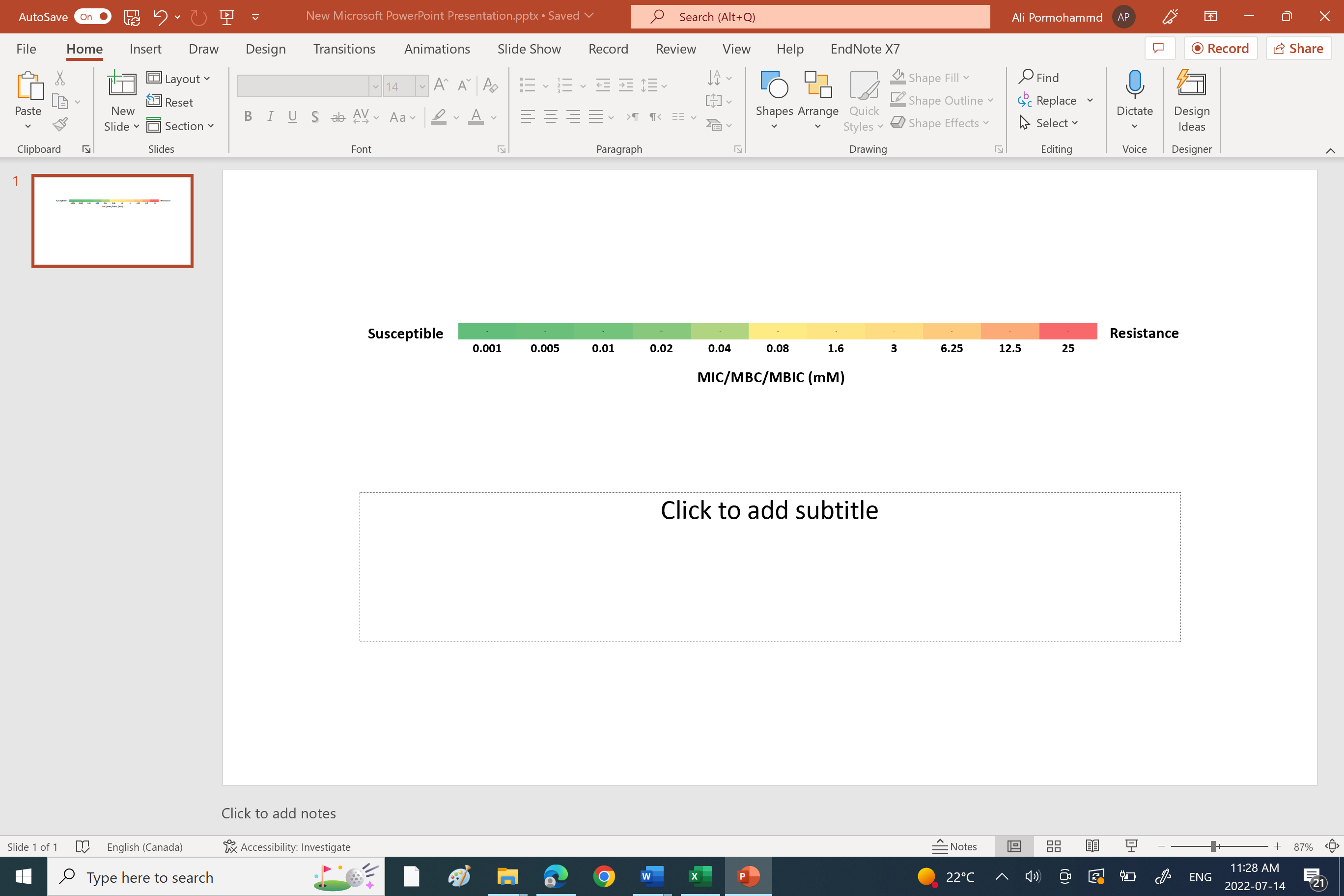


**Fig S1.** Minimum inhibitory concentration (MIC), Minimum bactericidal concentration (MBC), and Minimum biofilm inhibition concentration (MBIC) of diphenyl-urea antibiotics against MRSA clinical isolates.

**Fig S2. A. Hydrogen peroxide (H_2_O_2_) concentration.** Shows the H_2_O_2_  concentrations in the samples with the naked eye (down) and plate reader (upper). The standard, 1mM solution of hydrogen peroxide was serially diluted with double distilled (DD) water 1:2 for a total of 11 samples. DD water was used as the blank and working reagent (WR). **B.** **Free iron [(ferrous) Fe^+2^]** **concentration.** After 1 h treatment with selected components relative amount of ferrous was measured by OD. The naked eye (upper) Heatmap (down) is illustrated in the panels. **C.** **Reduced thiol (RSH) level.** Shows the RSH absorbance values in the samples with the naked eye (upper) and plate reader (down). 1mM solution of glutathione reduced oxidized serially diluted with Tris/HCl 1:2 for a total of 11 samples as a standard. The 50 mM Tris/HCl pH 8 and Ellman's reagent were used as the blank. One-way ANOVA with Bonferroni’s posttest, data not significant, treated groups in comparison with the untreated group.
